# Correspondence

**Published:** 1868-11

**Authors:** Paul D. Carpenter

**Affiliations:** East Mendon


### Correspondence.

East Mendon, October 19th, 1868.

Dr. Miner, Editor Buffalo Medical and Surgical Journal:

Dear Sir:—The following case, which occurred in my practice, being rather unusual, I submit it to you for publication, if you think it of sufficient interest:

Ella C——, aged 10 years, was, on Sunday, October 4th, after eating plentifully of fruit, attacked with colic, which was relieved by vomiting. The next day she had a slight fever, and some tenderness at the epigastrium, which was relieved by counter-irritation, bismuth, etc. Tuesday, and the three following days, she felt well, and only complained of an occasional cramping pain in the stomach. She was kept on light diet, the food being retained without uneasiness. On Saturday evening, six days after the first symptoms, she was seized with vomiting, accompanied with great thirst and paroxysmal pain over the stomach, over which, at times, the slightest touch would occasion extreme pain, and shortly after would bear quite firm pressure there without shrinking. Bismuth was vomited, but a small powder of leptandrin was retained, and allayed the vomiting for some time, after which it re-commenced, the fluid ejected from the stomach being of a greenish color, with clots of black colored blood, which seemed to be mixed with a thick gelatinous substance, of a lighter color. In the morning, she arose and walked, declining assistance. A short time after, the respiration became hurried, the extremities cold, and the pulse slow and weak; on being raised she suddenly expired. At the autopsy, eight hours after, the omentum was found very much thickened, with adhesion to the intestine, but with no appearance of vascularity. At one point was a small amount of pus, all the appearances showing a chronic peritonitis of some time standing. On opening the stomach, the mucous membrane was found to be in a softened gelatinous condition, with patches of black colored blood intimately mixed with it, evidently the same as that vomited in the morning. The finger could easily brush away the softened membrane, showing the submucous membrane beneath, in a normal condition.

The freedom from constant pain, must have been due, I think, to disintegration of the nervous filaments, which followed from the softening of the membrane, while the absence of any prostration would seem to be due to the short continuance of the disorder, and the freedom from constant pain, which, had it been present, would soon have exhausted the nervous strength.

PAUL D. CARPENTER, M. D.

